# The appropriate hybrid surgical strategy in three-level cervical degenerative disc disease: a finite element analysis

**DOI:** 10.1186/s13018-019-1502-5

**Published:** 2019-12-16

**Authors:** Y. M. Xie, Y. C. Zheng, S. J. Qiu, K. Q. Gong, Y. Duan

**Affiliations:** 1 Department of Spine Surgery, Zhujiang Hospital, Southern Medical University, No.253 Middle Industrial Road, Haizhu District, Guangzhou, 510280 Guangdong Province China; 2 Department of Spine Surgery, The Second People’s Hospital of Shenzhen, Shenzhen, 518037 Guangdong China

**Keywords:** Three-level cervical degenerative disc disease, Hybrid surgery, Finite element

## Abstract

**Objective:**

The purpose of this FE study was to analyze the biomechanical characteristics of different HS strategies used in the treatment of three-level CDDD (one-level CDA and two-level ACDF).

**Methods:**

We validated the FE model of an intact cervical spine established by transferring the data, collected by 3D CT scan, to the FE software ABAQUS and comparing these data with the data from published studies. Then, the FE model of hybrid surgery was reconstructed to analyze the range of motion (ROM), facet joint force, and stress distribution on an ultrahigh molecular weight polyethylene (UHMWPE) core.

**Results:**

The current cervical FE model was able to measure the biomechanical changes in a follow-up hybrid surgery simulation. The total ROM of the cervical HS models was substantially decreased compared with the total ROM of the intact group, and the M2 (C3/4 ACDF, C4/5 CDA, and C5/6 ACDF) model had the closest total ROM to the intact group, but the facet joint force adjacent to the treatment levels showed very little difference among them. The stress distribution showed noticeable similarity: two flanks were observed in the center core, but the inlay of M2 was more vulnerable.

**Conclusions:**

Through the comparison of ROM, the facet joint force after CDA, and the stress distribution of the prosthesis, we find that M2 model has a better theoretical outcome, especially in preserving the maximum total ROM.

## Background

Cervical degenerative disc disease (CDDD) has become a common condition causing pain and/or neurological deficit secondary to compression of the nerve roots or spinal cord [1, 2]. Anterior cervical discectomy and fusion (ACDF) is a standard surgical procedure for CDDD. This procedure aims to decompress the affected neural components, preserve intradiscal height, and provide mechanical stability [3]. However, an increase in motion, shear strain, and intradiscal pressure in adjacent vertebrae after ACDF has also been reported [4].

Cervical disc arthroplasty (CDA) is one of the most carefully studied and documented spinal procedures. CDA has been indicated to be a safe and effective alternative to ACDF. CDA can maintain physiologic motion, restore disc height and some viscoelastic properties, decrease the morbidity of fusion, and allow earlier return to function. In addition, CDA can mitigate the adverse biomechanical changes at adjacent vertebrae and consequently prevent the progression of adjacent segment degeneration (ASD) [5].

Some studies have examined the ability of prostheses to absorb vibrational and impact loads at adjacent segments, but cervical disc prostheses have little ability to provide viscoelastic properties anywhere near those of a normal hydrated disc. Therefore, the approved indication for CDA is restricted to single-level or two-level cervical disc disease. Although one prospective trial demonstrated efficacy and safety for three-level CDA, the study was off-label and should be considered experimental [6].

How to address three-level CDDD has become a problem. A novel hybrid surgery (HS) strategy consisting of ACDF and CDA was introduced to treat three-level cervical disease. Barbagallo et al. [7] was the pioneer in choosing the HS strategy for two-level and three-level CDDD. Despite the encouraging clinical outcomes of HS, few biomechanical tests have been used in the past to investigate HS strategies [8], and these strategies have lacked a detailed internal structural response to external loading. It is not yet clear which HS strategy represents the best choice or whether HS could be efficient and beneficial for three-level CDDD.

Mathematical models such as the finite element (FE) method can be applied to discover structural responses to external loading and, more importantly, to establish an internal structural response such as stress to external loading [9]. Although several models of the cervical spine have been demonstrated in recent studies, efforts to analyze the internal response, especially to evaluate three-level HS strategies, are lacking. Therefore, the purpose of this FE study is to target biomechanical analyses of the different HS strategies used in the treatment of three-level CDDD (one-level CDA and two-level ACDF).

## Methods

### FE modeling and validation

The C2–C7 region was reconstructed by a 3D CT scan of the cervical spine of a male subject (age 25, height 178 cm, weight 75 kg). The present study was approved by the ethical committee of Southern Medical University. Space intervals of 0.625 mm were set in the coronal CT images, and the participant in a neutral, unloaded position. Medical image processing software (Mimics 10.1, Materialise Inc., Belgium) was used to construct the geometry, and the data were later transferred to finite element software (ABAQUS 6.11.1, Simulia Inc., USA) to build the spinal components of the vertebrae using CT images.

**Fig. 1:**
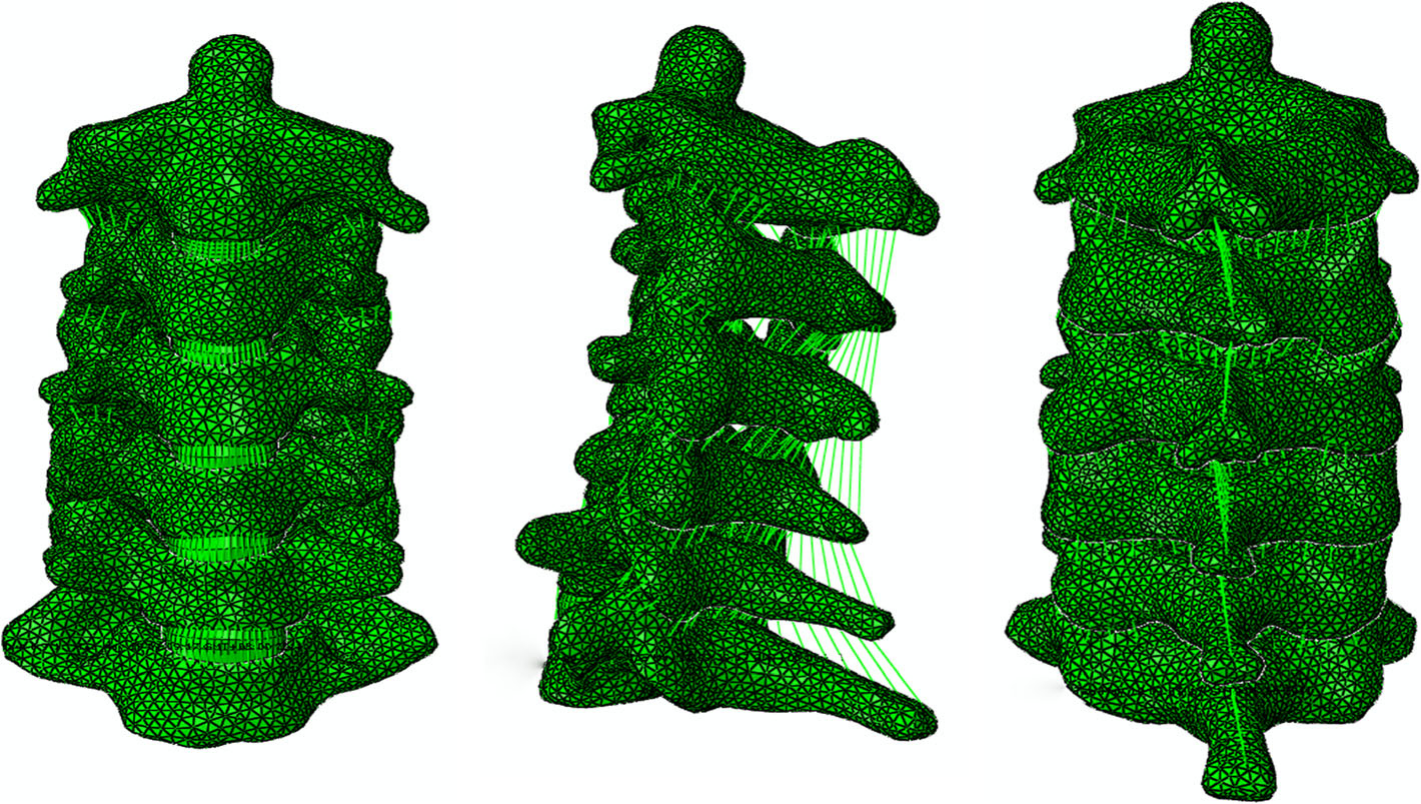
The intact FE model

The intact FE model consists of six vertebrae (C2, C3, C4, C5, C6, and C7), five intervertebral discs (C2–C3, C3–C4, C4–C5, C5–C6, and C6–C7), and all the important components of the cervical spine, such as the cortical bone, cancellous bone, intervertebral discs (consisting of the disc annulus and disc nucleus), and ligaments (Fig. 1). Solid elements were used for modeling vertebral bodies and posterior elements, but the material was described as isotropic. Shell elements were used for the cortical bone of the vertebral body, which is a very thin sheet of bone. A solid tetrahedral element was used for the cancellous part. A layer of shell with a thickness of 0.4 mm was divided into three regions (including two cartilage endplates and a cortical bone), covering the cancellous bone [10, 11]. To model the facet cartilage, the facet region was extracted from the geometry and enlarged into a solid volume by sweeping with a depth of 0.5 mm [12]. At a ratio of approximately 6:4, the intervertebral space was partitioned into annulus ground substance and the nucleus pulposus. A layer of net-like annulus fibers on the circumferential surface of the substance, which account for 19% of the volume of the annulus fibrosus, was constructed with an inclination between 15 and 45° with respect to the transverse plane [13].

**Fig. 2:**
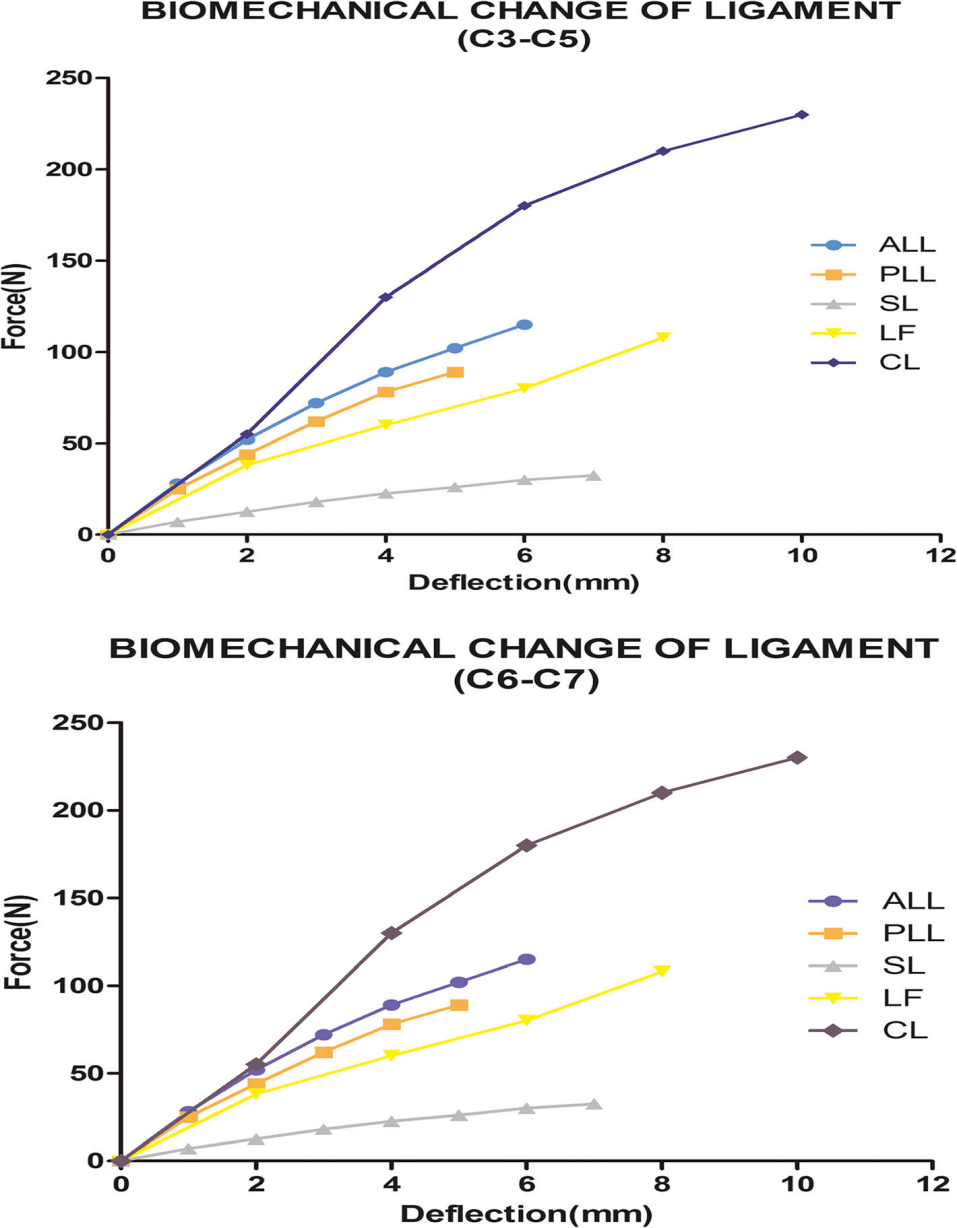
Biomechanical change of ligament (C3–C7)

Five different ligaments in the cervical spine, whose insertion points were chosen to mimic anatomic observations as closely as possible [14, 15], were rebuilt in the FE models as tension-only nonlinear connectors: the anterior longitudinal ligament (ALL), posterior longitudinal ligament (PLL), spinous ligament (SL), capsular ligament (CL), and ligamentum flavum (LF). Each spinal component represented the most commonly used values collected from the literature [16–20], and the material and mechanical properties are shown in Table 1 and Fig. 2.

**Table 1:** Material and mechanical properties of different parts used in the finite element model

Component	Young’s modulus (MPa)	Poisson’s ratio	Element type
Cortical bone and endplate	12,000.0	0.3	Triangular shell element
Cancellous bone	100.0	0.25	Tetrahedral element
Disc annulus	3.4	0.4	Hexahedral element
Disc nucleus	1.0	0.49	Hexahedral element
CoCr alloy	210,000.0	0.3	Tetrahedral element
UHMWPE	3000.0	0.3	Tetrahedral element
Ligament	Nonlinear tension only connector

With 74 N of axial compression superior to C2, static analysis was conducted by imposing 1.8 Nm of flexion-extension, lateral bending, and axial rotation movements. With all degrees of freedom constrained, the boundary condition was simulated by fixing the inferior surface of the C7 vertebra. The movements and the axial pre-compression forces were loaded onto C2. By using frictionless contact, the facet joints were simulated.

To verify the intact model, a comparison of the predicted results with those reported in the literature was performed.

### FE model hybrid surgery simulation

Based on a validated model of the aforementioned intact C2–C7 model, the data were then imported into the FE software package ABAQUS (v 6. 11.1) to build the surgery simulation models. Our study selected the Mobi-C cervical disc (a mobile-core ADR, LDR Medical, Troyes, France) as the CDA prosthesis, which consists of three components: a mobile-bearing device composed of two titanium plasma-sprayed and hydroxyapatite (HA)-coated cobalt-chromium alloy endplates and an ultrahigh molecular weight polyethylene (UHMWPE) mobile insert [21]. The material properties of the CoCr alloy and UHMWPE obtained from previous literature are also shown in Table 1.

To simulate the surgical procedure as closely as possible, the anterior longitudinal ligament at segments C3–C6 was excised. In addition, the annulus of the insertion area was removed by approximately 62% anteriorly, and the nucleus pulposus was completely removed. In addition, to maximize the bony contact area of Allograft and Mobi-C, the endplate of the CDA segment was partially removed.

Allograft and Mobi-C were implanted into C3/4, C4/5, and C5/6 for the HS models in three alternations. M1: C3/4 ACDF, C4/5 ACDF, and C5/6 CDA; M2: C3/4 ACDF, C4/5 CDA, and C5/6 ACDF; and M3: C3/4 CDA, C4/5 ACDF, and C5/6 ACDF. The models were designed to simulate the mid-long stage postoperatively; thus, the ACDF segments did take into account bone fusion.

### Biomechanical comparison

The same boundary and loading conditions were applied to the HS models. A precompression of 74 N was imposed on C2 in all simulations. At a pure moment of 1.8 Nm in all directions (flexion-extension, lateral bending, and axial rotation), the simulations were run for each model. The ROM was measured in the intact model and the HS models. Facet joint force and stress distribution in the Mobi-C prosthesis were compared among the different types of HS.

## Results

### FE model validation

**Fig. 3:**
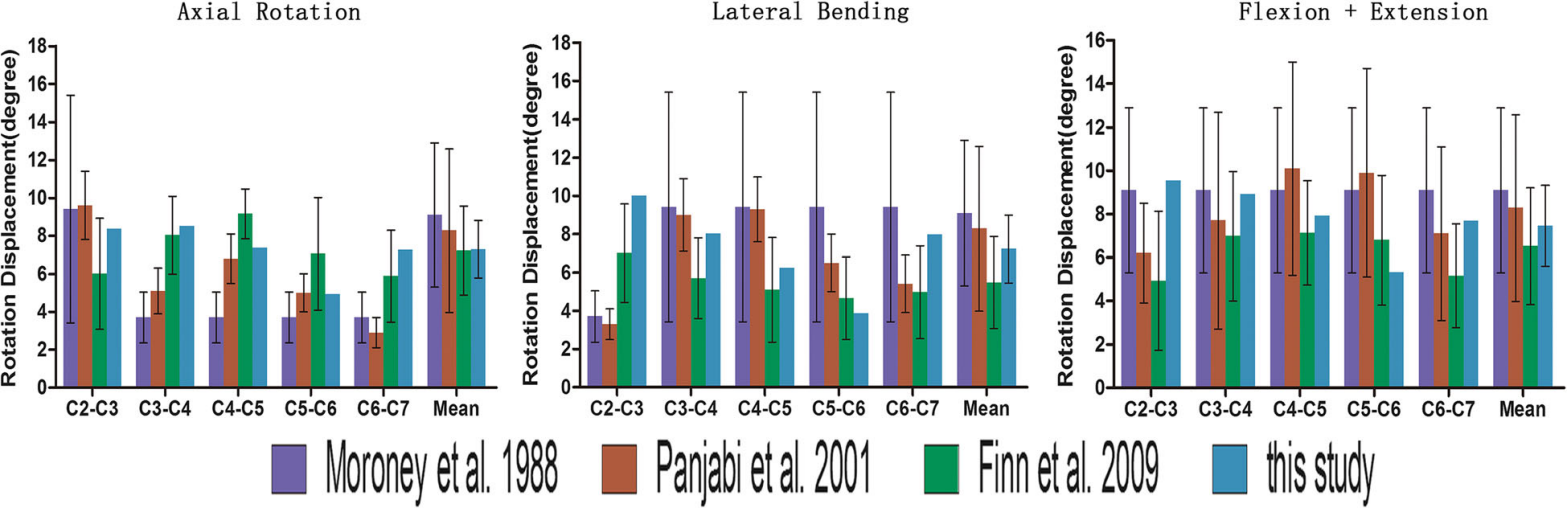
The details of the previous data used in the comparison (The rotation displacement comparsion of our study and the other three published studies)

The final intact model consisted of 130,429 elements and 30,181 nodes. Figure 3 shows the details of the previous data used in the comparison. The figure summarizes the comparison of the intersegmental responses between the intact model and previously published data under combined flexion-extension, lateral bending, and axial rotation conditions. All the predicted responses were well paired with the published data reported in previous studies [22–24]. Therefore, the current cervical FE model was able to demonstrate the biomechanical changes in a follow-up hybrid surgery simulation.

### FE model surgery simulation

**Fig. 4:**
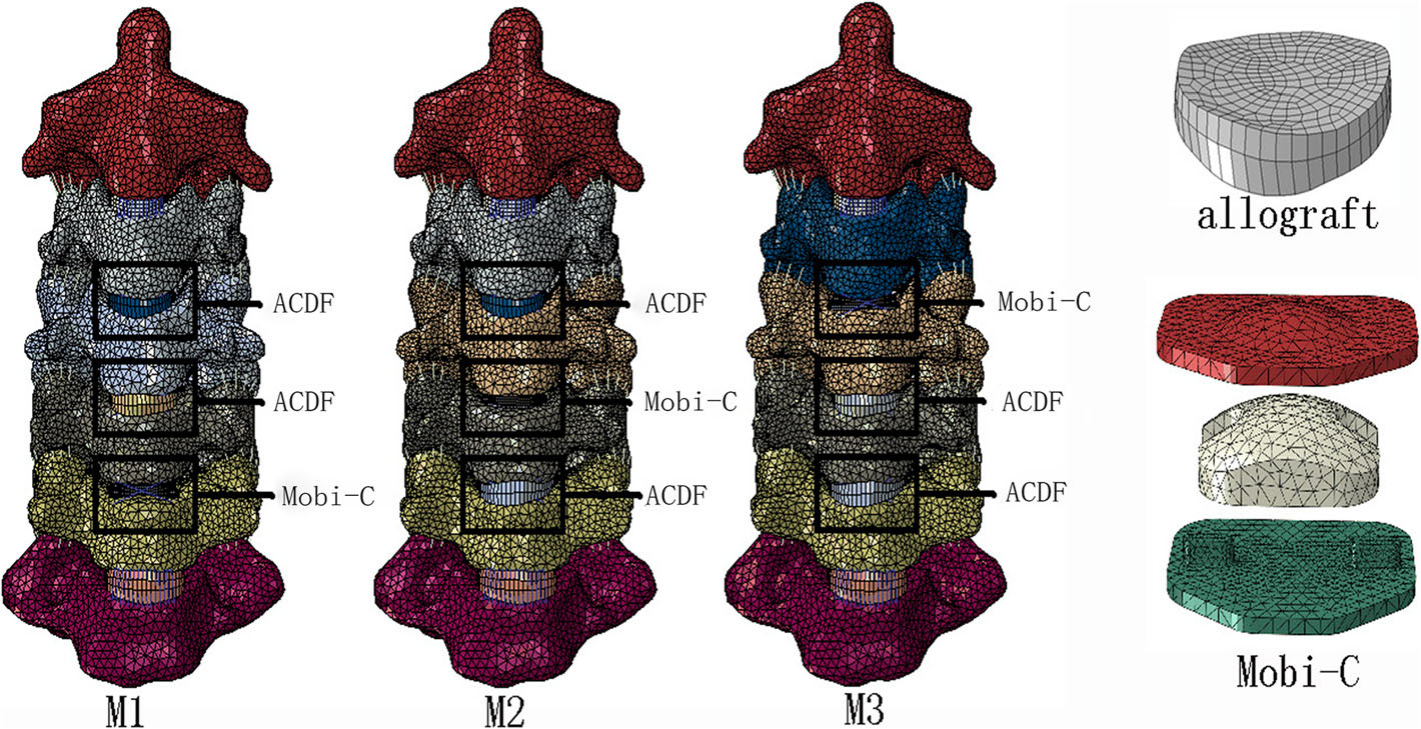
FE models with one-level CDA and two-level ACDF

Figure 4 illustrates the surgery-simulated FE models with one-level CDA and two-level ACDF. In the present study, Allograft and Mobi-C were implanted into arranged intervertebral spaces, which were assumed to be fully integrated. Their bone-bone or bone-prosthesis surfaces were simulated by imposing an ideal, rough behavior, thus preventing mobility.

### Intersegmental and total ROM analyses

Compared to that in the normal model, the intersegmental ROM of the C2/3 and C6/7 segments after hybrid surgery showed no noticeable differences, as presented in Fig. 4. However, the intersegmental ROM of the arranged CDA segments had noticeable differences compared to the previous intersegmental ROM.

**Fig. 5:**
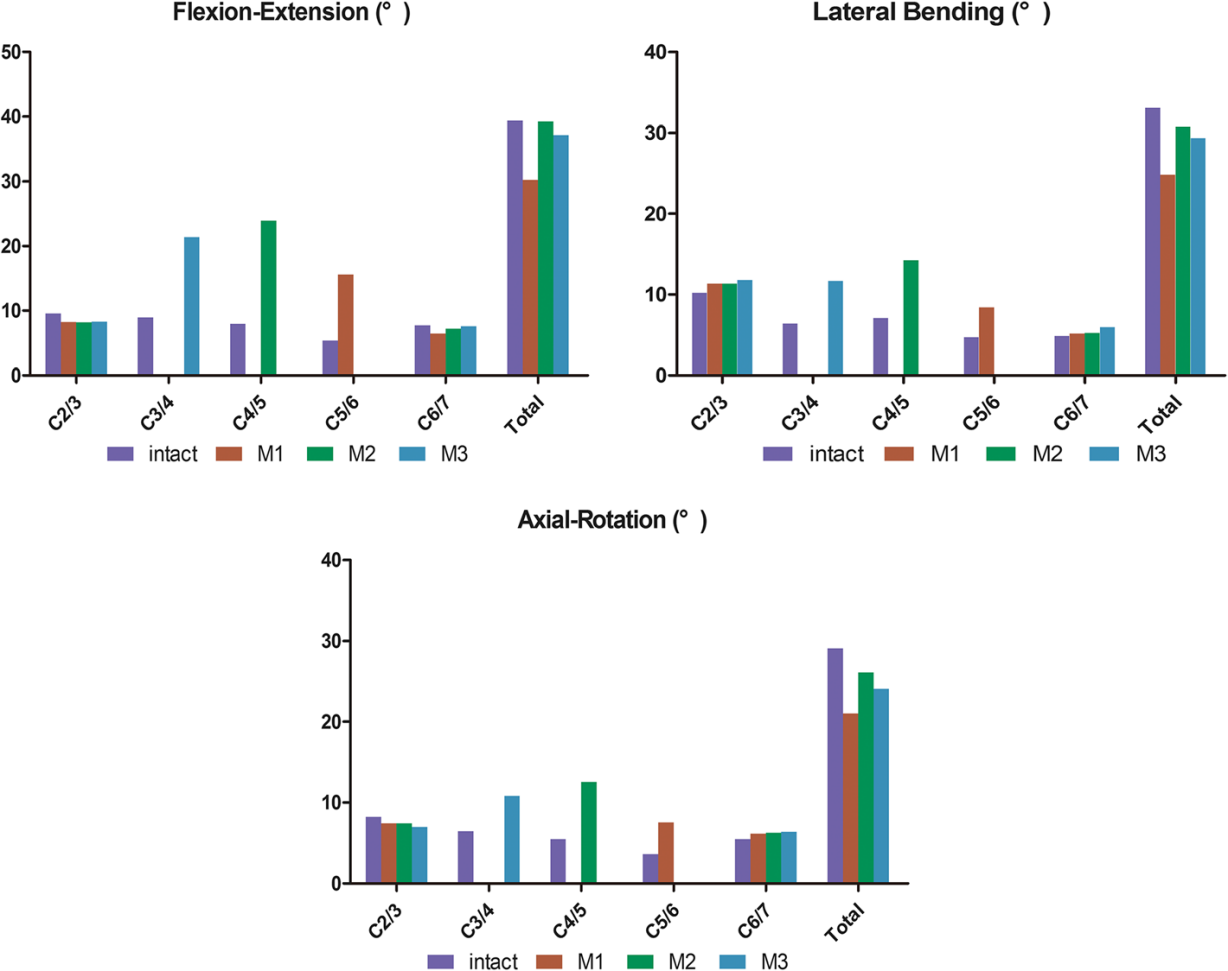
Range of motion of the segments under different conditions

Under flexion-extension, lateral bending, and axial rotation conditions, the total ROM of the intact and surgery-simulated FE models showed noticeable differences, which have important clinical significance. In the present study, the total ROM of the cervical HS models was substantially decreased compared with that of the intact group. However, the M2 model had the closest total ROM to the intact group, especially under flexion-extension and axial rotation conditions (Fig. 5).

### Facet joint force analyses

**Fig. 6:**
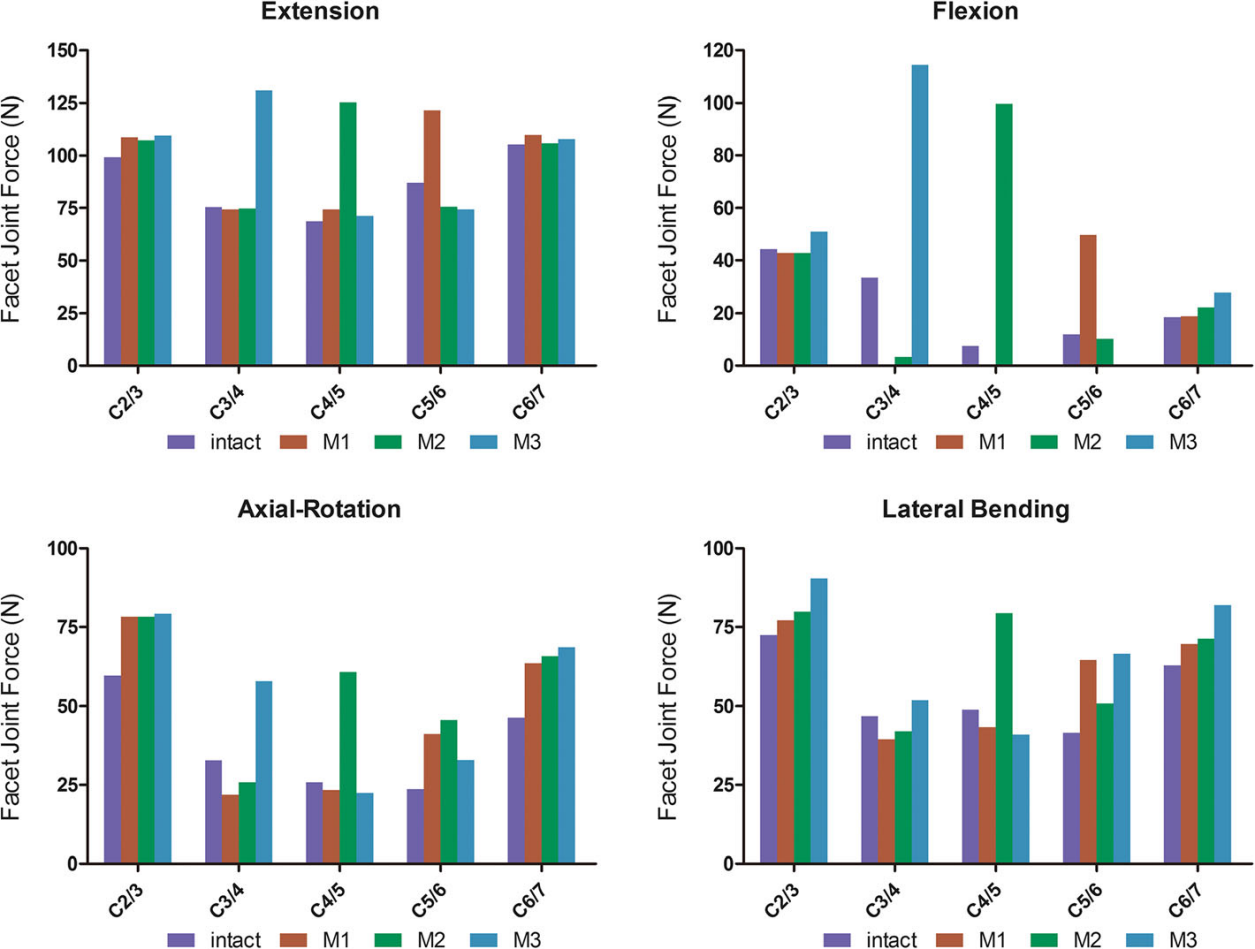
The facet joint force at each segment under different conditions

The facet joint force was measured at each segment. The facet joint force adjacent to the treatment levels (C2/3, C6/7) showed very little difference among the three HS groups compared with the intact group. However, the facet joint force at CDA segments was different at the corresponding segments in the intact cervical spine model (Fig. 6). As the histogram clearly shows, the facet joint force of CDA levels increased to some extent. The maximum increased range of facet joint force was measured under flexion conditions. The face joint force at the CDA segment of the HS models increased 3.3-fold in M1, 3.2-fold in M2, and 3.4-fold in M3, corresponding to the segment of the intact model. The maximal value of the facet joint force was obtained under extension conditions. The facet joint force was 121.43 N at the C5/6 segment in M1, 120.30 N at the C4/5 segment in M2, and 130.7 N at the C3/4 segment in M3.

### Stress distribution on the UHMWPE core

**Fig. 7:**
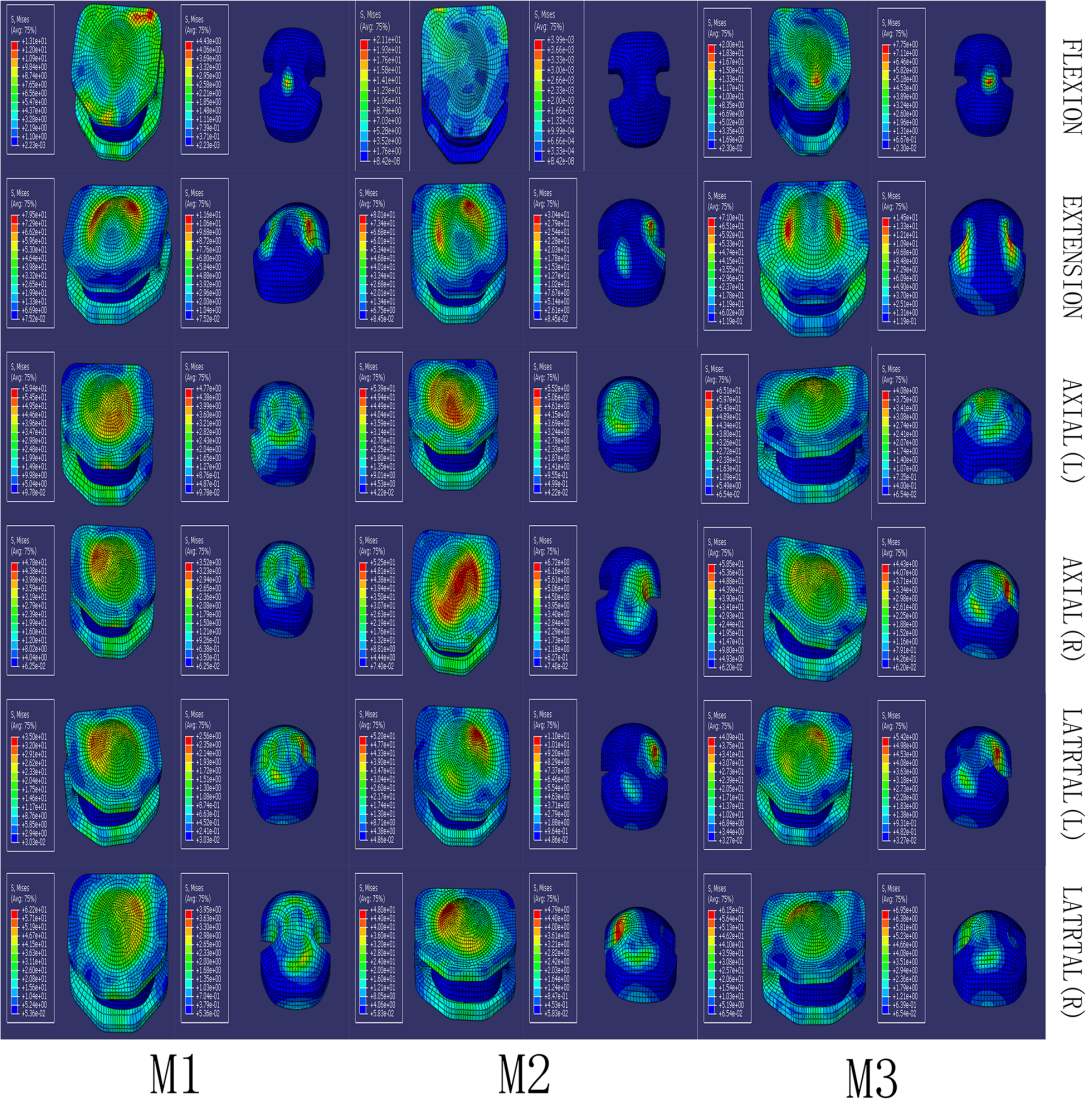
The stress distribution located at each CDA segment of HS models

The stress distribution located at each CDA segment in the HS models is presented in Fig. 7. Under flexion-extension, lateral bending, and axial rotation conditions, the stress distribution of the UHMWPE cores showed noticeable similarity, and the distribution was two flanks from the center core. The maximal value of stress was 11.6 MPa at the C5/6 segment in M1, 30.44 MPa at the C4/5 segment in M2, and 14.4 MPa at the C3/4 segment in M3.

## Discussion

Three-level CDDD is common in clinical practice [25], but surgeons are occasionally confronted with a patient requiring surgical treatment due to CDDD, involving multiple levels. Because of a paucity of data regarding three-level CDDD, the optimal management of this disorder remains controversial and case dependent. While ACDF has long been advocated for three-level CDDD, there is evidence supporting more substantial biomechanical effects in three-level fusion [26, 27]. The anterior approach may have a little more trauma than the posterior approach. There are still some annoying complications with the posterior approach such as axial pain and concomitant cervical kyphosis without fixation. The degree of degeneration in different levels is much variant among patients, some levels in some patients are still fit for arthroplasty surgery. HS strategy is a combination of replacement and fusion operation that can reduce the fusion segment and thus reach the goal of retaining as much ROM as possible, reducing the stress between the adjacent intervertebral disc and decreasing the unfusion rate.

To assess the ROM after three-level HS to find out how much ROM we can retain compares to the intact model, we chose to compare HS with intact cervical spine, because all degrees of freedom of C7 is constrained by the relative fixing inferior surface of the C7 vertebra. If the replacement segment is located at C6/7, it might affect the data. We should choose the segments of C3/4–C5/6 rather than C4/5–C6/7. In the present study, the ROM of segments C2/3 and C6/7 in the HS models confirmed an interesting phenomenon. The results indicated that there were no significant ROM changes in the segment adjacent to the operative segments compared to the intact group. Under flexion-extension, lateral bending, and axial torsion conditions, the total ROM of the HS models decreased to some extent in the present study. This result is consistent with the results from a study by Cho et al. [28]; the use of a cadaveric biomechanical test showed that two-level ACDF could decrease the entire ROM, and two-level CDA and hybrid ACDF/CDA did not show significant changes in the adjacent-level ROM. However, our study showed an encouraging result in that the M2 model had the closest total ROM to the intact group, especially under flexion-extension and axial torsion conditions. In this regard, the results of this study may indicate how to choose an optimal HS strategy in terms of preserving the maximum total ROM.

The increased ROM found at the CDA segments is probably caused by the excision of the ALL and the anterior parts of the annulus. Furthermore, the increased ROM may have resulted in increased stress of the facet joint force in the implanted segment [29]. An increase in the facet joint force after CDA was reported to accelerate degeneration in implanted segments [30], and subsequent alterations in cervical spine biomechanics result in hypertrophy of the ligamentum flavum and facet joint laxity. Some studies have indicated that the increased facet joint force in implanted segments might lead to the degeneration of new segments [31]. Lee et al. [20] analyzed the biomechanical changes in Mobi-C after CDA. The ROM in the CDA segment increased during flexion (33%), extension (56%), lateral bending (35%), and axial torsion (105%). The facet joint force increased by 210% in both fixed and mobile core models. In our study, the facet joint force at the CDA segments was different from the force at the corresponding segments in the intact cervical spine model. As the histogram clearly shows, the facet joint force of the CDA segments increased to some extent. However, the facet joint force adjacent to the treatment levels (C2/3, C6/7) was hardly different among the HS groups compared with the intact group. Based on the results described above, we believe that his procedure has no adverse effect on the facet joints of adjacent segments but on the facet joints before operation due to the increase in the facet joint force after CDA, which may be regarded as a risk factor that leads to new segment degeneration.

Over the last decade, some cervical devices have gained US Food and Drug Administration (FDA) approval [32]. We classified the cervical devices as constrained or mobile types according to the type of prosthesis implanted. Mobi-C belongs to the mobile type of devices; despite the fact that its insertion point slightly misses the center, it tends to have a biomechanical impact on the facet joint because the UHMWPE core of the prosthesis is translocated, while the pressure on the facet joint is high [33]. Our result is also in agreement with the results of the study mentioned above, showing increased pressure on the UHMWPE core in Mobi-C with a mobile core. However, our study showed that the maximal force value of the UHMWPE core was increased in the M2 strategy compared with the HS strategies. This finding indicates that the UHMWPE core may wear easily in the M2 strategy.

The present study, focusing on the changes in the ROM, facet joint force, and UHMWPE core, aimed to analyze the biomechanical performance after three-level HS. The conclusions are summarized as follows: (1) HS does not affect the ROM or facet joint force of the adjacent segment in the prevention of ASD, (2) the results of total ROM show that the M2 strategy is the best HS strategy in terms of preserving the maximum total ROM, (3) the ROM and the facet joint force in the CDA segments clearly increase, and (4) the maximal force value of the UHMWPE core is increased in the M2 strategy compared with the other strategies.

## Conclusion

In our study, we try to find the best theoretical combination in these three models by analyzing the FE simulation. As the discussion above, through the comparison of ROM, the facet joint force after CDA, and the stress distribution of the prosthesis, we find that M2 model has a better theoretical outcome, especially in preserving the maximum total ROM. In clinical practice, we choose the hybrid surgery according to the strict surgical indications. If patients can be unconstrained to choose all the three combinations, M2 is recommended according to our theoretical results. If the replacement segment is the exclusive selection, we should choose the corresponding combination.

## Limitation

In the study, there are still several limitations. Though we have conducted some research about the cervical three-level HS simulation, there are much further improvements that shall be carried out. First, if the EF model can include the entire spine and surrounding muscles, the biomechanical simulation of the entire model will be greatly improved. The additional soft tissues such as muscles can imitate the dynamic effect of the active and passive contraction that might make our work much more integrated and authentic. Second, the EF model only reflects the biomechanical state of a particular time period and cannot reflect the continuous dynamic and biomechanical state of the whole time period. Finally, the results of the EF research can only theoretically support the researchers’ hypothesis, and the reliability of the results will ultimately need to be confirmed by cadaver specimens and clinical studies.

## Data Availability

The datasets generated and/or analyzed during the current study are available from the corresponding author on reasonable request.

## Abbreviations

3D: Three-dimensional
ACDF: Anterior cervical discectomy and fusion
ALL: Anterior longitudinal ligament
ASD: Adjacent segment degeneration
CDA: Cervical disc arthroplasty
CDDD: Cervical degenerative disc disease
CL: Capsular ligament
CT: Computed tomography
FE: Finite element
HA: Hydroxyapatite
HS: Hybrid surgery
LF: Ligamentum flavum
PLL: Posterior longitudinal ligament
ROM: Range of motion
SL: Spinous ligament
UHMWPE: Ultra-high molecular weight polyethylene
